# TURP-like syndrome without surgery: severe dilutional hyponatraemia following prolonged sterile water bladder washouts for radiation cystitis: a case report

**DOI:** 10.1093/omcr/omag150

**Published:** 2026-08-11

**Authors:** Hafiz Awais Javed, Daisy Beard, Christopher Launchbury, Rehan Masood, Amna Zeeshan, Haris Marath

**Affiliations:** Department of Diabetes and Endocrinology, West Suffolk NHS Foundation Trust, Bury St Edmunds, Suffolk, IP33 2QZ, United Kingdom; Department of Diabetes and Endocrinology, West Suffolk NHS Foundation Trust, Bury St Edmunds, Suffolk, IP33 2QZ, United Kingdom; Department of Diabetes and Endocrinology, West Suffolk NHS Foundation Trust, Bury St Edmunds, Suffolk, IP33 2QZ, United Kingdom; Department of Diabetes and Endocrinology, West Suffolk NHS Foundation Trust, Bury St Edmunds, Suffolk, IP33 2QZ, United Kingdom; Department of Diabetes and Endocrinology, West Suffolk NHS Foundation Trust, Bury St Edmunds, Suffolk, IP33 2QZ, United Kingdom; Department of Diabetes and Endocrinology, West Suffolk NHS Foundation Trust, Bury St Edmunds, Suffolk, IP33 2QZ, United Kingdom

**Keywords:** hyponatraemia, radiation cystitis, sterile water bladder washout, continuous bladder irrigation, TURP syndrome, dilutional hyponatraemia

## Abstract

Severe dilutional hyponatraemia is a recognised complication of transurethral resection of the prostate (TURP syndrome) caused by systemic absorption of hypotonic irrigation fluid, but similar events outside the operative setting are rarely reported. We describe a 77-year-old White male with radiation cystitis who underwent 12 days of sterile water bladder washouts alongside continuous 0.9% saline bladder irrigation for persistent haematuria. Serum sodium declined from 132 to 112 mmol/L and improved only after bladder washouts and irrigation were discontinued. Cystoscopy confirmed radiation cystitis with mucosal ulceration. The clinical course strongly suggests that systemic absorption of electrolyte-free sterile water caused the profound hypotonic hyponatraemia, whereas concurrent absorption of isotonic saline contributed to extracellular volume expansion. This case highlights that prolonged sterile water bladder washouts may produce a TURP-like syndrome in the absence of surgery. Early recognition, serial electrolyte monitoring and prompt source control are essential to prevent potentially life-threatening complications.

## Introduction

Hyponatraemia is the most common electrolyte disorder in hospital practice and is associated with increased morbidity and mortality [1]. TURP syndrome classically results from systemic absorption of hypotonic irrigation fluid during endoscopic prostate surgery [2, 3]. Although irrigation-fluid absorption is well recognised during operative urological procedures, similar complications associated with prolonged non-operative bladder washouts are rarely reported. Radiation cystitis is characterised by chronic inflammation, ischaemia, friable neovascularization and urothelial ulceration, which disrupt the urothelial permeability barrier [4]. Previous reports have described fatal fluid overload, hyperchloraemic metabolic acidosis and haemolysis associated with bladder irrigation [5–7], but severe dilutional hyponatraemia remains rarely reported outside the operative setting. We report a case of severe dilutional hyponatraemia following prolonged sterile water bladder washouts in a patient with radiation cystitis, producing a TURP-like syndrome in the absence of surgery.

**Figure 1:**
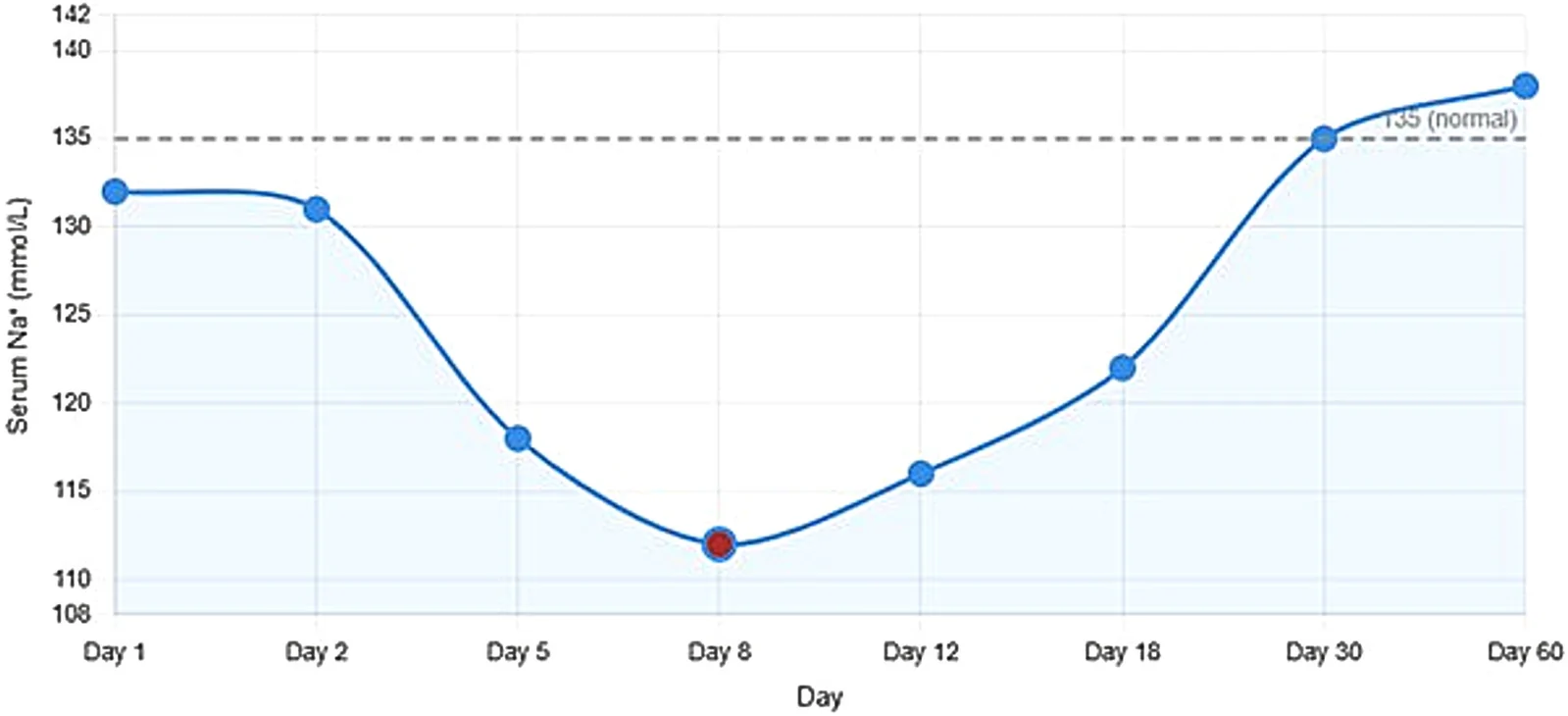
Trend in serum sodium concentration during prolonged sterile water bladder washouts. Serum sodium progressively declined from 132 mmol/L on admission to a nadir of 112 mmol/l on day 8 during prolonged sterile water bladder washouts and continuous bladder irrigation. Serum sodium subsequently recovered following cessation of bladder washouts and bladder irrigation.

**Table 1:** Clinical timeline with daily washout and CBI status.

Day	Na^+^ (mmol/L)	Hb (g/l)/Hct (L/l)	Sterile water washouts	CBI (0.9% NaCl)	Key events
1	132	132 g/l/0.385	Yes	Yes	Admission; catheterisation; CT urogram; IV 0.9% NaCl commenced
2	131	—	Yes	Yes	Haemodynamically stable
5	118	—	Yes	Yes	Progressive hyponatraemia
7	115	91 g/l/0.24	Yes	Yes	Hb decline; no major haemorrhage source identified
8 (nadir)	112	79 g/l/0.21	Yes	Yes	1.8% hypertonic saline commenced
9	113	—	Yes	Yes	Serum osmolality 240 mOsm/kg; urine indices uninterpretable (catheter contamination)
10	115	67 g/l/0.183	Yes	Yes	Hb/Hct nadir; no major haemorrhage; TFTs, cortisol and renal function normal; 1 unit of packed red blood cells transfused
12	116	76 g/l/0.21	Discontinued	Discontinued	Cystoscopy: radiation cystitis with mucosal ulceration; diathermy; washouts and CBI discontinued
18	122	81 g/l/0.21	None	None	Continuing recovery
30	135	82 g/l/0.24	None	None	Outpatient; normonatraemia
60	138	84 g/l/0.25	None	None	Sustained normonatraemia

## Case report

A 77-year-old White male with a history of prostate adenocarcinoma treated with external beam radiotherapy was admitted with macroscopic haematuria and clot retention. On admission, he was haemodynamically stable and clinically euvolaemic. Serum sodium was 132 mmol/l. Renal function, thyroid function and morning cortisol were normal. There was no clinical evidence of heart failure, chronic liver disease, nephrotic syndrome or significant proteinuria. CT urography demonstrated an intravesical clot without upper urinary tract pathology.

Following urethral catheterisation, daily manual bladder washouts using sterile water were performed. Continuous bladder irrigation with 0.9% sodium chloride (approximately 20–25 L/day) was commenced because of ongoing haematuria. No hypotonic intravenous fluids were administered during the admission. Serum sodium progressively declined from 132 mmol/L to 118 mmol/L by day 5 and reached a nadir of 112 mmol/l on day 8 (Fig. 1). Although the patient remained neurologically intact, hypertonic (1.8%) saline was commenced in accordance with local institutional practice because of the severity of the biochemical hyponatraemia. However, serum sodium showed minimal improvement while sterile water bladder washouts continued.

Serum osmolality was 240 mOsm/kg, confirming true hypotonic hyponatraemia. Urine collected on day 9 from the catheter drainage bag (urine osmolality 287 mOsm/kg, urine sodium 71 mmol/l) was considered uninterpretable because a urinary creatinine concentration of < 1.1 mmol/l confirmed contamination by irrigation fluid. Accordingly, urinary sodium and osmolality were excluded from mechanistic interpretation.

By day 12, the patient had developed peripheral oedema and clinical hypervolaemia. During the same period, haemoglobin fell from 132 g/l to 67 g/l and haematocrit from 0.385 to 0.183 L/l. Although ongoing haematuria secondary to radiation cystitis likely contributed to the decline in haemoglobin, there was no evidence that haemorrhage alone could account for the magnitude of the fall. The parallel reductions in haemoglobin and haematocrit suggested substantial haemodilution secondary to systemic fluid absorption. One unit of packed red blood cells was transfused after the haemoglobin reached 67 g/l. Normal reticulocyte count and lactate dehydrogenase argued against haemolysis as a significant contributor.

Flexible cystoscopy performed on day 12 demonstrated radiation cystitis with mucosal ulceration. Diathermy was undertaken, and both bladder washouts and continuous bladder irrigation were discontinued. Histopathological examination demonstrated acute-on-chronic inflammatory infiltrates, ulceration and patchy haemorrhage without significant urothelial atypia or evidence of malignancy. Serum sodium subsequently increased to 122 mmol/l by day 18, 135 mmol/l at one month and 138 mmol/l at two months. No further hypertonic saline, fluid restriction or vasopressin antagonist therapy was required (Table 1).

## Discussion

This case demonstrates severe hypotonic dilutional hyponatraemia associated with prolonged sterile water bladder washouts and continuous bladder irrigation in a patient with radiation cystitis. The temporal relationship between prolonged sterile water bladder washouts, progressive hyponatraemia and recovery after cessation strongly supports systemic absorption through the damaged urothelium. These findings suggest that systemic absorption of electrolyte-free sterile water was the principal mechanism underlying the profound hypotonic hyponatraemia, whereas concurrent absorption of isotonic 0.9% saline likely contributed to extracellular volume expansion and hypervolaemia without directly lowering serum sodium.

Radiation cystitis is characterised by chronic inflammation, ischaemia, neovascularization and mucosal ulceration, disrupting the urothelial permeability barrier. Classical TURP syndrome results from systemic absorption of hypotonic irrigation fluid during transurethral surgery. Although our patient did not undergo surgery, the underlying mechanism appears analogous, with prolonged exposure of ulcerated bladder mucosa to electrolyte-free sterile water resulting in profound hypotonic hyponatraemia.

Serum osmolality confirmed true hypotonic hyponatraemia. The patient was initially clinically euvolaemic despite progressive hyponatraemia but subsequently developed peripheral oedema and hypervolaemia, illustrating that progressive irrigation-fluid absorption may initially mimic euvolaemic hyponatraemia before extracellular volume expansion becomes clinically evident. Although ongoing haematuria contributed to the decline in haemoglobin, the parallel reduction in haematocrit, development of hypervolaemia, and normal reticulocyte count and lactate dehydrogenase levels suggest that haemodilution from systemic fluid absorption also made a substantial contribution. One unit of packed red blood cells was transfused after the haemoglobin reached 67 g/l. Urinary sodium and osmolality could not be interpreted because irrigation-fluid contamination rendered the urine sample unreliable. Although concomitant syndrome of inappropriate antidiuresis could not be formally excluded, the overall clinical course strongly supports irrigation-fluid absorption as the dominant pathophysiological mechanism.

Hyponatraemia improved only after bladder washouts and irrigation were discontinued despite several days of hypertonic saline therapy, highlighting that source control rather than supportive therapy was the definitive intervention. Earlier cessation of sterile water bladder washouts may have limited the severity of hyponatraemia. As the hyponatraemia evolved over more than 12 days, careful monitoring during recovery was essential to minimise the risk of osmotic demyelination syndrome. Neither desmopressin nor relowering therapy was required.

Limitations include the inability to quantify irrigation-fluid absorption, contamination of urinary indices preventing formal exclusion of concomitant syndrome of inappropriate antidiuresis, the confounding effect of ongoing haematuria on interpretation of haemodilution, and the inherent limitations of a single case report.

This case highlights that prolonged sterile water bladder washouts in patients with radiation cystitis may result in severe hypotonic dilutional hyponatraemia through systemic absorption across a damaged urothelial barrier, producing a TURP-like syndrome in the absence of surgery. Where bladder washouts are required, sterile water should be avoided and patients requiring prolonged bladder irrigation should undergo serial electrolyte monitoring to facilitate early recognition and prompt source control of this potentially life-threatening complication.

## References

[1] Spasovski G, Vanholder R, Allolio B. et al. Clinical practice guideline on diagnosis and treatment of hyponatraemia. Eur J Endocrinol 2014;170:G1–47. 24569125 10.1530/EJE-13-1020

[2] Mamoulakis C, Skolarikos A, Schulze M. et al. Results from an ongoing prospective randomised controlled study comparing bipolar with monopolar transurethral resection of the prostate. Eur Urol 2009;56:633–41. 10.1016/j.eururo.2012.10.003 23102675

[3] Demirel I, Ozer AB, Bayar MK. et al. TURP syndrome and severe hyponatremia under general anaesthesia. BMJ Case Rep 2012;2012:bcr2012006879. 10.1136/bcr-2012-006899 PMC4543890 23166168

[4] Zwaans BMM, Lamb LE, Bartolone SN. et al. Pathophysiology and management of radiation cystitis. Res Rep Urol 2019;11:97–107.31114765

[5] Di Paolo M, Bugelli V, Di Luca A. et al. Bladder irrigation and urothelium disruption: a reminder apropos of a case of fatal fluid absorption. BMC Urol 2014;14:91. 25410651 10.1186/1471-2490-14-91 PMC4247597

[6] Latif A, Thirumalareddy J, Sood A. et al. First reported case of hyperchloremic non-anion gap metabolic acidosis in a patient undergoing continuous bladder irrigation for hemorrhagic cystitis. Cureus. 2020;12:e11927. 33489544 10.7759/cureus.12132 PMC7813527

[7] Bell MD . Sudden death due to intravascular hemolysis after bladder irrigation with distilled water. J Forensic Sci 1992;37:1401–6. 1402764

